# Regional climatic changes and their impact on the level of avalanche hazard in East Kazakhstan

**DOI:** 10.1016/j.heliyon.2025.e41807

**Published:** 2025-01-09

**Authors:** Olga Petrova, Natalya Denissova, Gulzhan Daumova, Yelena Ivashchenko, Evgeny Sergazinov

**Affiliations:** a School of Earth Sciences, D. Serikbayev East Kazakhstan Technical University, Ust-Kamenogorsk, 070001, Kazakhstan; b Department of Information Technology, D. Serikbayev East Kazakhstan Technical University, Ust-Kamenogorsk, 070001, Kazakhstan; c Department of Research Activities, D. Serikbayev East Kazakhstan Technical University, Ust-Kamenogorsk, 070001, Kazakhstan; d Department of Support and Information Technology, D. Serikbayev East Kazakhstan Technical University, Ust-Kamenogorsk, 070001, Kazakhstan

**Keywords:** Data analysis, Entity relationship diagram, Climate change, Avalanches, Monitoring system, East Kazakhstan, Continental climate

## Abstract

The article examines the territory of East Kazakhstan, where a sharply continental climate prevails with hot summers, cold and snowy winters. The mountainous regions of East Kazakhstan are represented by the Kalba, Altai and Saur-Tarbagatay ranges, they are surrounded by rolling plains. The highest points are at 3000–4500 m. On average, the heights are in the range of 900–1400 m. Despite the low heights in the mountainous area, the problem of avalanche safety is acute in the region. At the same time, the situation is complicated by not always predictable weather events, the frequency of which is increasing every year. These include heavy precipitation, sometimes combined with a sharp warming in winter, and the changing wind regime of the territory. To identify regional climate changes and its connection with the avalanche-prone situation in the region, the study analyzed meteorological data from weather stations located directly near avalanche prone locations over the past 23 years since 2001, as well as data from observations of avalanche-prone areas since 2005 and information on registered spontaneous avalanches from 2013 to the present. This study is the first in the East Kazakhstan region, which presents the results of a comprehensive analysis of data on 497 avalanche-prone sites, of which 325 sites pose a threat to life and infrastructure. 10 most dangerous sites have been selected for detailed study. The analysis of climate data was carried out based on information from 7 weather stations. The article discusses the main climatic changes in the region, including an increase in air temperature, an increase in precipitation and a change in wind conditions. Data from weather stations and snow measuring routes covering the period since 2005 have been used to develop probabilistic avalanche forecasts. The use of statistical methods and the analysis of the relationships between meteorological parameters (temperature, precipitation, wind speed) made it possible to build models predicting avalanche-prone situations. Data on spontaneous avalanches were analyzed for five sites (Bogatyrevskaya site, Prokhodnaya, Sogornoye-Barlyk, Tainty and Pikhtovka). Based on these data, equations of dependence of temperature, wind and precipitation have been developed, which improves the accuracy of forecasting avalanche risks. An analysis of the data in the Statistica program showed a significant relationship between sudden warming, increased wind speed and precipitation, which precedes avalanches. Regression equations and the approximation confidence coefficient for the average values of the studied parameters are obtained. The results of the study make it possible not only to establish patterns, but also to propose effective methods for monitoring and forecasting avalanche hazard in the region. According to the data analysis, regional features of climate change in East Kazakhstan were identified, and a comparison was made with previously known works on Kazakhstan. The interrelation of climatic characteristics with avalanche hazard in the region is shown. The results obtained in the study will help us to better understand the regional manifestations of climate change. An important task for further forecasting of avalanche activity is the correct design of the avalanche collection database. The authors identified information objects (entities). An ontological database model (Entity Relationship Diagram) is constructed. Based on it, a database has been created for a system for monitoring and forecasting avalanche activity in the East Kazakhstan region.

## 1 Introduction

Climate change is one of the most important issues on the global environmental agenda. The entire planet is facing the global consequences of climate change. Studying the manifestations of climate change in individual regions is also an important task for their sustainable development. Regional manifestations of climate change may be specific to different regions of the planet.

The works of many climate scientists are devoted to global climate change. For example, the average temperature for one year is consistently higher than the average temperature for the period of 150 years for the Central Asian countries [1]. For Kazakhstan, the change in the average annual temperature has been studied since 1865.

Under global warming, changes in meteorological factors show significant variability in their spatial distribution and temporal trends [2].

Global climate change also has a significant impact on various natural processes, including avalanche hazard. Numerous studies show that global warming leads to changes in meteorological conditions, which, in turn, affects the stability of snow cover and the likelihood of avalanches [3].

The impact of climate change on the formation and characteristics of avalanches is manifested in a decrease in snow cover, an increase in the number of heavy snowfalls and an increase in air temperature. These changes lead to wet snow avalanches due to increased snow moisture and a sharp rise in temperature [4]. Climate change affects the area and timing of snow cover [5,6]. Precipitation, air temperature, and snow conditions can change significantly as the climate warms, leading to significant changes in avalanche risk [7].

The impact of climate change on snow cover is manifested through noticeable regional differences [8]. In particular, it should be noted that climate change has markedly increased global temperatures over the last century, thereby changing regional snow cover characteristics [9,10]. Global warming changes snowfall patterns in many parts of the world. The periods of snowfall and snow melt are also changed along with rising global temperatures [11]. There is a correlation between snowfall and air temperature in colder regions (high latitudes or high altitudes), which is associated with increased atmospheric moisture content. Warmer temperatures increase the amount of water vapor in the atmosphere, which contributes to a potential increase in snow volume if temperatures remain low enough for snow to fall [12].

The impact of climate change on snow conditions in Poland from 1981 to 2020 shows the sensitivity of snow cover to climate warming and its decline in large lowland areas [13].

Tracking changes in temperature and precipitation in the Central Tien Shan in the Kunse River valley from 1967 to 2021 showed that winter temperatures increased significantly by 0.32 °C per decade. This was accompanied by more intense snowfalls. There was a decrease in the duration of snow cover by 4.77 days over the period under review, which is reflected in the later onset of winter cover and earlier melting. At the same time, average and maximum snow depths have increased, as have peak snow water equivalents, indicating more frequent years with very little or heavy snowfall. It has been established that climate warming and extreme weather conditions, which are commonly observed in the central Tien Shan Mountains, have changed the physical characteristics of the snowpack, significantly affecting the timing, scale and types of avalanches. Increased air temperatures shortened the duration of snow cover and led to earlier snow melting, which in turn caused an earlier active period of snowmelt and wet avalanches [14].

The impact of climate change on avalanche activity in one of the most susceptible areas in Norway was studied [15]. The researchers analyzed long-term weather data from seven official weather stations, three of which had observations spanning 120 years (1896–2015). Statistical analysis revealed a consistent trend towards rising air temperatures and increasing precipitation, especially over the past 30 years. A study of the amplitude and frequency of monthly precipitation showed an increase in precipitation, particularly noticeable in the last three decades, with precipitation projected to increase in March and February. The authors found that an increase in monthly precipitation and an increase in temperature in winter can lead to a general increase in the frequency of snow avalanches.

Having analyzed the above scientific studies, it should be noted that changes in the characteristics of snowfall depend on climate, atmospheric circulation, meteorological and ground conditions, which, in turn, affect avalanche activity.

Thus, many years of research experience in predicting and identifying the causes of snow avalanches shows that the avalanche formation process is influenced not only by the above factors themselves, but also by their complex combination.

Many of the authors' studies focused on high mountains with developed tourism infrastructure, while significantly less research was devoted to lower altitudes, although these are areas where winter snowfall can have more severe impacts. Moreover, these areas are more likely to have greater impacts on urban and rural communities, as such communities are much more likely to be located at lower altitudes.

For example, the problem of avalanche hazard in low mountain ranges was studied using the example of the Giant Mountains [16], whose height is 1603 m, located in the northeastern part of the Czech Republic [17]. Since the mid-2000s, due to climate change, the number of wet snow avalanches due to melting snow has increased. The authors found that the most significant parameters influencing avalanches are the maximum and minimum air temperature, snow depth, wind speed and direction, and the amount of precipitation. An analysis of data from 1979 to 2020 showed an increase in air temperature during the winter season of 1.8 °C since 1979. The most important variables explaining avalanche activity were snow depth, precipitation depending on threshold temperature, amount of snowfall, and wind speed.

No previous study has examined avalanche risk in East Kazakhstan caused by global warming using a comprehensive analysis of meteorological parameters. Thus, the relationship between climate change and meteorological characteristics affecting the occurrence of avalanches is being studied for the first time for this region and is an urgent problem on a global scale. This study focuses on regions with similar climates to further explore changes in meteorological conditions in the context of global warming and their impact on avalanche risk, complementing global research in this area.

Understanding the relationship between meteorological factors and avalanches is a key to effectively predicting and preventing avalanche situations. Research around the world confirms the importance of meteorological factors in the formation of avalanche conditions. Similar trends were found across different geographic regions, although there were some differences in the impact of specific parameters.

Numerous studies have been conducted in the Alps and other mountainous regions of Europe identifying the relationship between temperature, precipitation and wind speed with avalanche activity. It has been established that high temperatures, intense precipitation and strong winds can significantly increase the risk of avalanches [18,19].

To determine the snow cover class, other authors [20] used a binary system based on three climatic variables: wind, precipitation, and air temperature. They have developed a classification system for seasonal snow covers based on unique textural and stratigraphic characteristics. It included the sequence of snow layers, thickness, density, crystal morphology and grain properties in each layer.

There is known work on atmospheric circulation, which has an important influence on avalanche activity in the region, generally determining weather conditions in the mountains, affecting the formation of avalanches [21]. The authors analyzed synoptic atmospheric conditions on days with different avalanche activity. The results showed that the increase in avalanche activity is associated with atmospheric circulation, which contributes to large amounts of precipitation, increased wind speed and air temperature near Spitsbergen (Norway).

Studies in North America [22,23] and the Alps [24] showed the relevance and necessity of studying meteorological data for avalanche forecasting using databases.

For mountainous regions of North America, such as the Rocky Mountains and Colorado Mountains, researchers have also looked at the influence of meteorological factors on avalanche hazard. Particular attention was paid to changes in the humidity and temperature of the snow cover [25].

Previous studies in the above-mentioned area have shown that the likelihood of avalanche situations is influenced by meteorological factors, which include air temperature, precipitation, wind speed, as well as snow characteristics, such as its density and structure [26].

Using the example of mountainous areas of the western part of the United States, the influence of various variables on extreme avalanche events is assessed. It was found that high water levels in the snowpack and heavy snowfall most closely correspond to days of increased avalanche hazard [27]. This study included daily data on the number and volume of avalanches, as well as climate data such as maximum and minimum temperatures, total snow depth, amount of new snowfall, snow water equivalent (SWE) and precipitation. Multivariate statistical analysis was also used to determine which climatological variables are most useful for predicting extreme avalanche conditions.

The connection between climate change and the occurrence of avalanches has also been studied using the example of India [28]. This study indicates that the formation of snow avalanches is influenced by meteorological conditions, such as air temperature, wind speed, and atmospheric pressure.

In the following studies, meteorological factors such as total precipitation and daily maximum temperatures are examined during time periods from 24 h to 7 days before the onset of avalanche activity [[29], [30], [31]].

Collection and analysis of meteorological data is a basic requirement for the development of avalanche hazard monitoring systems. Among the developed monitoring systems, one can note the comprehensive web-based avalanche hazard monitoring system created by French researchers [32]. The authors recommend using three data sets for forecasting: avalanche records (Enquete Permanente sur les Avalanches, EPA), avalanche maps (Carte de Localization des Phénomenes d'Avalanche, CLPA) and a collection of hazard data for populated areas. The data is integrated into a common database, ensuring full interoperability between all types of avalanche records: digitized geographic data, avalanche characteristics, eyewitness reports, photographs, hazard and risk levels.

A variety of approaches are used to predict and assess avalanche risk, including the use of numerical models. Numerical models allow us to estimate the parameters of an avalanche, including its speed, volume and impact pressure, based on the physical characteristics of the snow cover and the topography of the area.

The use of models to estimate the pressure of an avalanche impact is critical for the design of protective structures, infrastructure planning in mountainous areas and improving the safety of tourist routes [33]. For example, in the work of the authors [34], transients associated with the pressure of an avalanche impact are considered. For this purpose, a three-dimensional non-Newtonian model based on the Navier-Stokes equations was developed. To analyze transients, the avalanche impact pressure was measured on a specially equipped obstacle measuring 1 m in height and 0.65 m in width, designed to study high-density wet snow. The experiments and simulations made it possible to compare the experimental and calculated values of the avalanche impact pressure, which provides opportunities for assessing the load on structures in conditions of a dense avalanche flow.

The database plays a key role in verifying and improving avalanche dynamics models, especially in conditions of high wet snow density. Proper understanding of avalanche flow parameters is crucial to protecting people and infrastructure in mountainous regions. For example, the Coulomb dynamic coefficient of friction of snow is an important parameter for evaluating avalanche characteristics such as descent range, speed, force and transverse propagation in hilly terrain. In the work of the following authors [35], this coefficient is defined as the ratio of the shear force to the normal component of the avalanche force. In a study based on 32 measurements carried out from 2017 to 2020, the authors presented a new database including the values of shear forces, normal avalanche forces and the obtained values of the dynamic coefficient of friction.

The albedo of the snow cover is one of the key parameters influencing climatic processes in the atmosphere–underlying surface system [36]. In mountainous areas with seasonal snow cover, this indicator varies significantly both in spatial and temporal aspects [37]. Even a slight decrease in snow albedo can lead to an increase in the absorption of solar radiation [38], which, in turn, has a significant impact on hydrological processes, including changes in the timing and intensity of snowmelt.

The authors of the following study [39] suggested that a homogeneous medium simultaneously absorbs, emits and dissipates radiation. They developed a technique for simultaneous evaluation of three key parameters: scattering albedo, thermal conductivity parameter and surface emissivity. To do this, the inverse method was used, which combines the lattice Boltzmann method, the finite volume method and a genetic algorithm. The direct method used the finite volume method to calculate radiation characteristics, and the lattice Boltzmann method was used to solve the heat balance equation.

Thus, avalanche risk forecasting and monitoring are relevant in the context of climate change, which affects the stability of snow cover and the frequency of avalanches.

The avalanche-prone territories of East Kazakhstan have become the object of research by Kazakh and Russian scientists. For example, one of the Kazakhstani researchers [40], dealing with avalanche safety issues, found that snowstorm-type avalanches prevail in the mountains of East Kazakhstan in all high-altitude zones. In addition, he suggested that avalanche formation is possible in all mountainous regions of East Kazakhstan, except for intermountain basins.

Data on avalanches in the East Kazakhstan region were also presented in Russian publications [41,42] and in the Kazakhstan Atlas of Natural and Man-made Hazards and Emergency Risks in the Republic of Kazakhstan [43].

According to Blagoveshchenskiy [44], avalanches are also observed in mountainous areas along the eastern border of Kazakhstan. Among them, the Kazakh researcher identifies the Altai, Kalba, Saur-Tarbagatai ranges located on the territory of East Kazakhstan. In his work, the author pays attention to meteorological parameters that affect the occurrence of avalanches.

The novelty of this study lies in an integrated approach to the analysis of avalanche-prone areas of East Kazakhstan, including both spatial distribution and a detailed study of the influence of climatic factors on avalanche activity. The importance of this work is justified by the extreme climatic conditions of the region and the significant change in long-term climatic characteristics due to global processes. This leads to such weather manifestations as sudden temperature fluctuations, intense precipitation, and strong winds. As a result, frequent avalanches occur, threatening people and infrastructure, including tourist sites and highways.

The purpose of the study is to analyze meteorological data, identify the relationship between climate change and avalanche risk, and develop a model of probabilistic forecasts to improve the accuracy of avalanche risk prediction in East Kazakhstan.

**Fig. 1:**
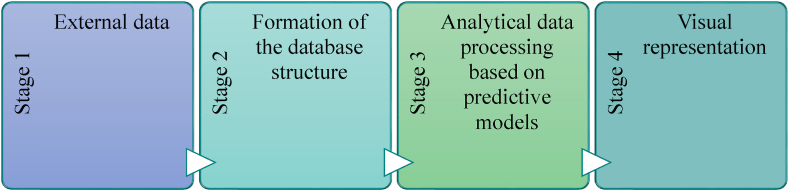
Key aspects of the study.

The key aspects of the study are shown in Fig. 1.

## 2 Study area

An avalanche hazard monitoring system is being developed by the authors for East Kazakhstan. The area of the East Kazakhstan region is 97,800 km^2^. This region has unique natural conditions, where various natural complexes are in a relatively small area and the pole of continentality is located. It is in East Kazakhstan that the point furthest from all the oceans in the world is located. This location determines the sharply continental climate of the region. But it is distinguished by its diversity between steppes and semi-deserts in the south and southwest, and mountainous regions in the north and northeast. The region's climate is characterized by significant temperature fluctuations, both seasonal and daily, with cold and snowy winters and hot and dry summers. The climatic features of the mountainous regions of the eastern part are formed by the Saur-Tarbagatay, Kalba and Altai Mountain ranges. There are eternal glaciers here, which also influence the microclimate of the territory. Winters in the region are cold and long, with minimum temperatures reaching −54°С.

**Fig. 2:**
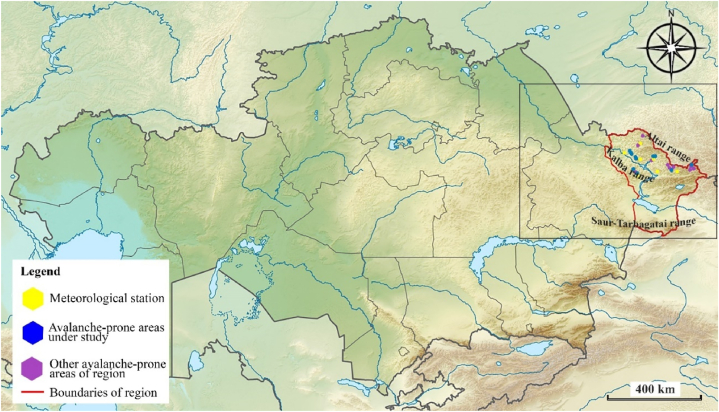
Location of the research area on the map of Kazakhstan.

**Fig. 3:**
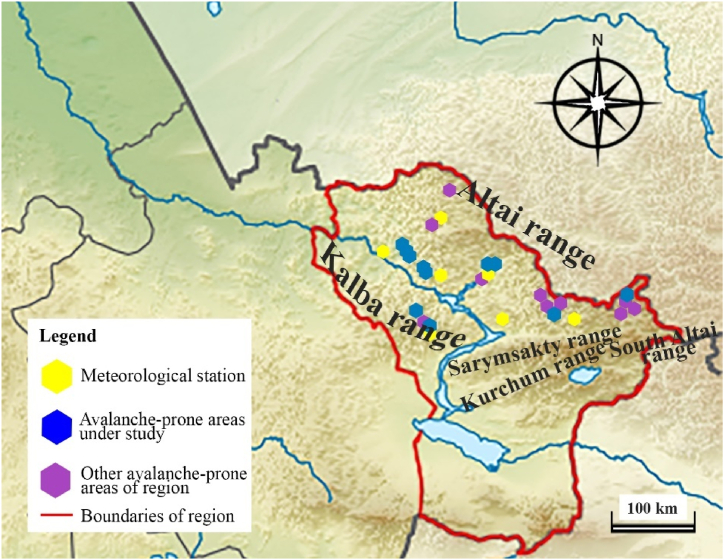
Research area on the map of East Kazakhstan.

The area of study is shown in Fig. 2, Fig. 3. In Fig. 2, the area of Fig. 3 is shown by a rectangle.

The distribution of annual precipitation throughout the region is very uneven; the largest amount falls in mountainous and foothill areas. Here, extreme weather conditions: heavy rainfall, sudden warming in winter, strong winds are increasingly causing spontaneous avalanches. At the same time, many people live and work in the mountainous regions of East Kazakhstan; there is a tourism infrastructure and an extensive network of roads. All this is in areas influenced by avalanche hazard areas.

In this regard, an important step for the region is the development of an avalanche hazard monitoring system. Such a system will be a hardware and software complex for analyzing avalanche conditions and transmitting appropriate alerts. The strategy for creating this system includes the collection of various historical, archival and current data on meteorological parameters, the condition and morphology of slopes, including remote sensing data and the creation of digital twins, data on spontaneous avalanches, and their processing using direct and inverse modeling methods, and machine learning.

**Fig. 4:**
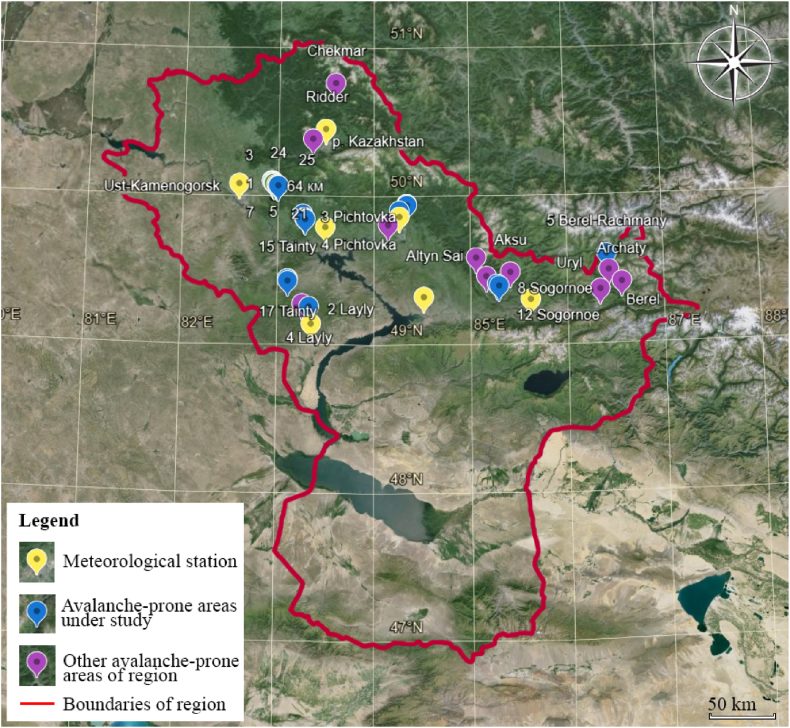
Location of avalanche areas in East Kazakhstan.

The authors examined data on 497 avalanche-prone areas in the region, of which 325 areas threaten objects and people's lives. The location of the most dangerous areas is marked in Fig. 4.

**Fig. 5:**
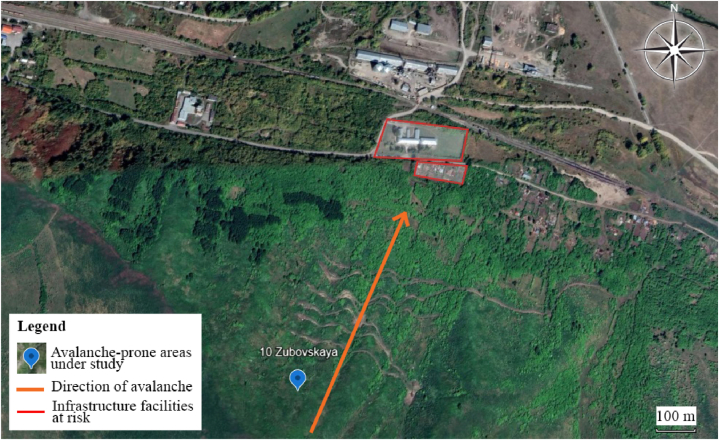
The relative position of the studied avalanche areas and weather stations.

**Fig. 6:**
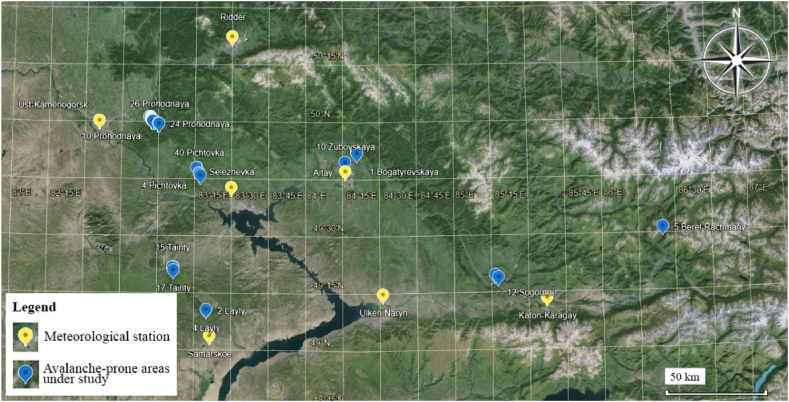
Location of infrastructure facilities near an avalanche-prone area.

**Fig. 7:**
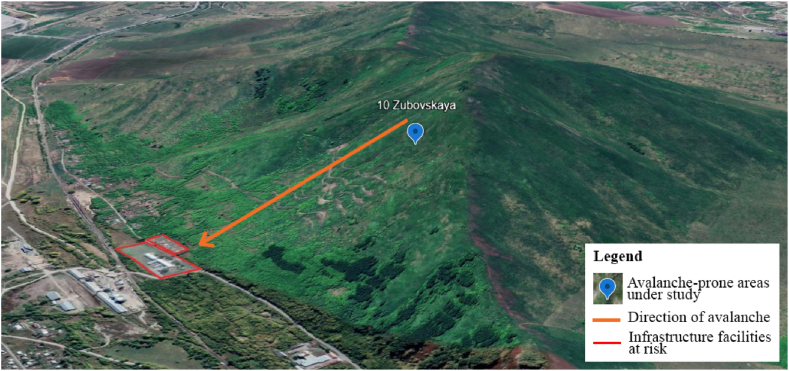
Location of residential buildings and schools at the foot of Mount Zubovskaya.

The 10 most significant avalanche-prone areas were selected for detailed study when developing a monitoring system. In addition, the analysis of climate data was carried out specifically for this region at 7 weather stations located near the selected 10 sites. Fig. 5 shows the location of weather stations and avalanche areas. They were determined after reviewing and digitizing data from meteorological observation logs on snow measuring routes. In these areas, the largest number of spontaneous avalanches occurred, and the most vulnerable infrastructure facilities are located. For example, in the Zubovsk village at the foot of Mount Zubovskaya, residential buildings and the school are in the avalanche zone. This avalanche-prone area is shown in Fig. 6, Fig. 7.

**Fig. 8:**
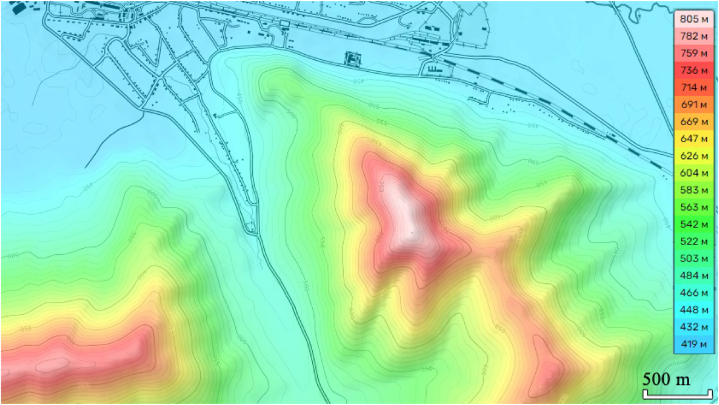
Elevations of Mount Zubovskaya.

When considering climate change and avalanche hazard in the region, it should be noted that on average the altitudes of the mountainous regions in question are in the range of 900–1400 m, surrounded by hilly plains. The highest points are at 3000–4500 m, but near such mountains there are practically no infrastructure facilities, and no risk situations are created. For example, the height of Mount Zubovskaya is 805 m, and the height difference from the foot to the top is 350–400 m (Fig. 8).

## 3 Materials and methods

The data discussed in the article is collected from two main sources. These are data from seven weather stations of the national hydrometeorological service of Kazakhstan « Kazhydromet » [45]. Observational history, available in digital format, covers the period from 2001 to the present. For analysis, the data was taken at the end of the winter period – 03/31/2024. For two weather stations, digital data is only available from 2008. This period was sufficient to assess climate change in the region.

The second source of data is digitized data from observation logs on snow measuring routes in the East Kazakhstan region since 2005 and emergency information data from the State Institution « Kazselezaschita » on avalanches since 2013 [46]. This work was carried out by the authors to create a database for the avalanche hazard monitoring system being developed. Snow route data includes the average and maximum snow depth recorded for each avalanche area. As well as the air temperature during the day. These data are supplemented by observers with descriptions of weather conditions, such as cloudiness, wind, precipitation, without measuring the absolute values of these quantities.

To understand regional climate changes, the processing of climate data was carried out using the Excel program, making forecasts of indicators using polynomial dependencies of the 2nd degree.

The data set is shown in Table 1.

**Table 1:** Characteristics of the data used for analysis.

№	Data type	Data subtype	Max	Min	Average	Data acquisition period, year
1	RSE « Kazhydromet»					
	Air temperature, ° C	Average per day per year	4,4	1,3	3,2	2001-2023
		Average per day in winter	−5,2	−12,1	−7,8	2001-2023
	The amount of precipitation, mm	Average per day per year	1,7	0,9	1,3	2001-2023
		Average per day in winter	1,7	0,7	1,1	2001-2023
		The amount for the year	621	319	485	2001-2023
		The amount for the winter	302	131	204	2001-2023
	Relative humidity of the air, %	Average per day per year	72	63	68	2001-2023
		Average per day in winter	76	70	73	2001-2023
	Lack of air saturation	Average per day per year	6,05	3,75	4,71	2001-2023
		Average per day in winter	1,64	1,06	1,43	2001-2023
	Snow height, cm	Average per day per season	37	14	25	2001-2023
	Wind speed, m/s	Average per day per year	2,4	1,8	2,2	2001-2023
		Average per day in winter	2,4	1,6	2,1	2001-2023

2	SI « Kazselezashchita»					
	Snow height, cm	On snow measuring route	148	5	77	2005-2023

3	SI « Kazselezashchita»					
	Avalanches	Number of avalanches	142	17	65	2013-2024
		Volume of snow in avalanches, m^3^	593680	67675	220500	2013-2024

4	SI « Kazselezashchita»					
	Avalanche site	Bogatyrevskaya site, Prokhodnaya, Sogornoye-Barlyk, Tainty Pikhtovka				2013-2023
	Avalanche date	07.01.2023				2013-2023
		09.01.2019				
		25.11.2021				
		23.01.2020				
		25.12.2015				

Based on the analysis of data from meteorological stations and snow measuring routes, probabilistic avalanche forecasts are proposed, which makes it possible to predict avalanche-prone situations with greater accuracy. The analysis methodology is based on statistical data collected since 2005, which makes it possible to identify patterns of meteorological characteristics and develop an effective model for predicting avalanche risks.

To study the possibility of making probabilistic forecasts of avalanches, data on meteorological conditions and spontaneous avalanches in the Bogatyrevskaya site, Prokhodnaya, Sogornoye-Barlyk, Tainty and Pikhtovka. To assess the relationships between these variables, statistical methods of analysis were used, such as data processing in the Statistica program [47].

Information from all sources of meteorological data has been compiled into a single digital database, based on which an algorithm for analyzing these parameters will be developed in relation specifically to the East Kazakhstan region. Meteorological data are not the only important components of it. During the study of avalanche-prone areas in the East Kazakhstan region, the following information entities were identified: area, avalanche area, avalanche collection, meteorological data, morphological type, type of slope exposure, type of vegetation cover, avalanche collection vegetation, degree of avalanche hazard, device, observation parameter, data observations, preventative descents, self-dismounting's.

A database based on the MySQL database management system was created to store the collected data [48]. Google Earth Pro was used to form the cartographic basis of project [49].

**Fig. 9:**
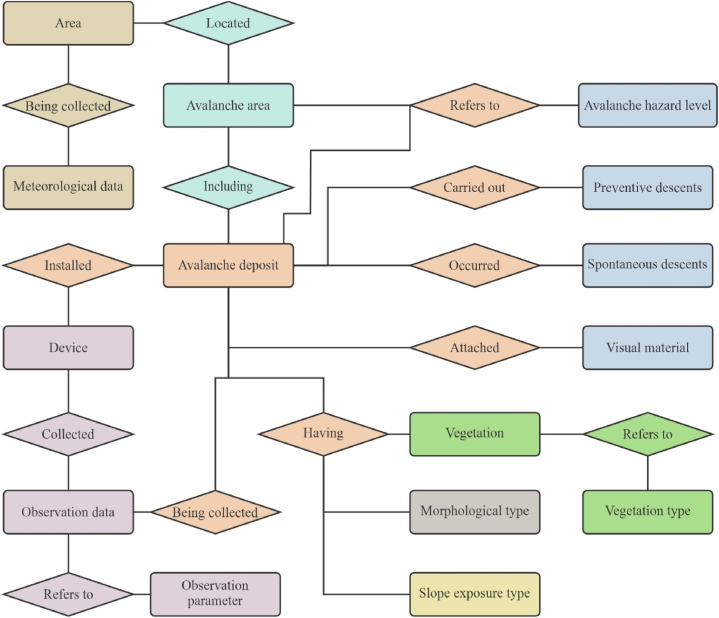
Entity Relationship Diagram of the database.

To understand the interaction between previously identified information entities, Fig. 9 shows an Entity Relationship Diagram. The diagram shows the information objects selected for research and the connections between them.

## 4 Results and discussion

### 4.1 Climate change

**Fig. 10:**
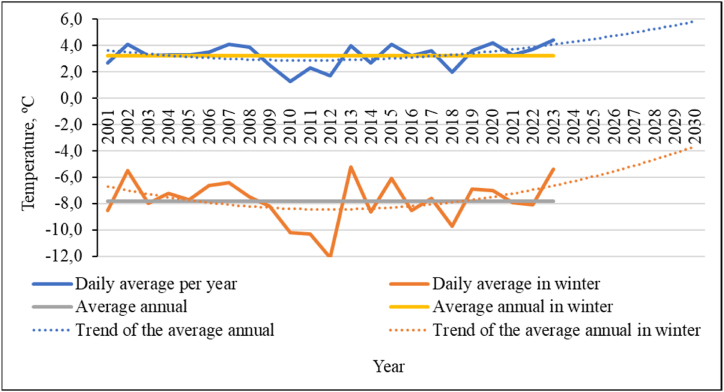
Average statistical air temperature data for the study area and forecast for their changes.

Analysis of meteorological parameters for the area where avalanche-prone areas are located showed changes that have occurred over a long period. Trend lines were constructed using a second-degree polynomial relationship. Temperature trends are consistent with global changes and indicate a gradual increase in air temperature in the area (Fig. 10). Despite the 2012 minimum, general trends continue, and the winter of 2023–2024 was the warmest for this region over the entire observation period. Also, according to previous studies [50], the summer of 2012 turned out to be abnormally warm in Kazakhstan, but at the same time the winter was extremely cold throughout the entire territory of Kazakhstan. It was also noted that global warming not only does not exclude significant negative temperature anomalies at the regional level but may contribute to their intensification. The minimum in the graph of Fig. 10 is associated with this. At the same time, the five warmest years in Kazakhstan for the period before 2012 were included in the list of the ten warmest years in the entire globe.

The regression equations and the confidence value of the approximation (R^2^) of the obtained trend values are given in equation (1) for the average values for the year and equation (2) for the winter period:

(1) $$\begin{array}{c} y=0,008x^{2}-0,17x+3,8\\ R^{2}=0,16 \end{array}$$

(2) $$\begin{array}{c} y=0,015x^{2}-0,35x-6,4\\ R^{2}=0,12 \end{array}$$

**Fig. 11:**
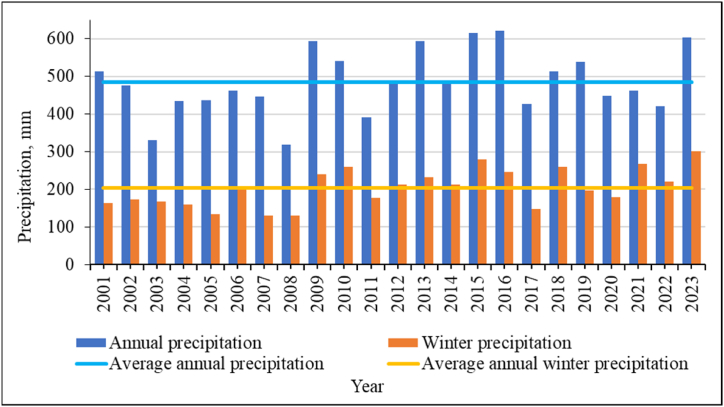
Average annual precipitation and average winter precipitation for the study area.

Fig. 11 shows data on the amount of precipitation in this area. As can be seen from the graph, there is a gradual increase in the amount of precipitation, both average annual, and an increase in the amount of precipitation in winter. Both indicators consistently exceed the average values for the study period since 2009.

**Fig. 12:**
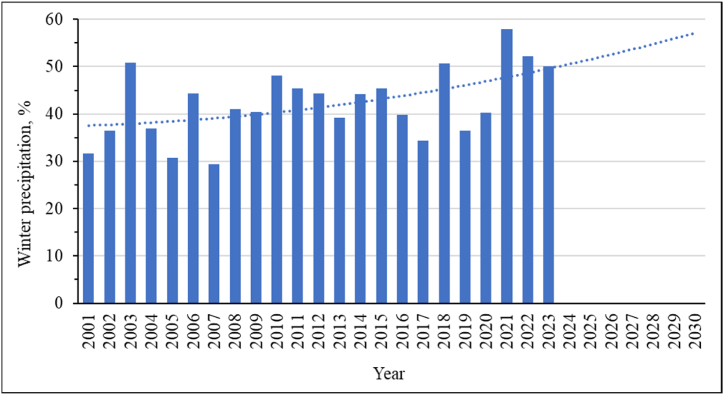
Winter precipitation as a percentage of average annual precipitation for the study area.

It should be noted that the climate of East Kazakhstan is characterized by snowy winters. But if previously the greatest amount of precipitation fell during the warm season, then recently there has been a steady trend of increasing precipitation in winter (Fig. 12). This is due to the general climatic trends in the region. The air temperature is rising, which leads to a drier summer. But at the same time, the air temperature rises in winter, which contributes to greater saturation of the air with water vapor and more precipitation. This means that hot summers become even more arid, and winters are even more snowy. The trend line of the polynomial dependence of the second degree, shown in Fig. 12, confirms this trend.

The regression equation and the confidence value of the approximation (R^2^) of the obtained trend values are given in equation (3):

(3) $$\begin{array}{c} y=0,018x^{2}+0,10x+37,5\\ R^{2}=0,26 \end{array}$$

**Fig. 13:**
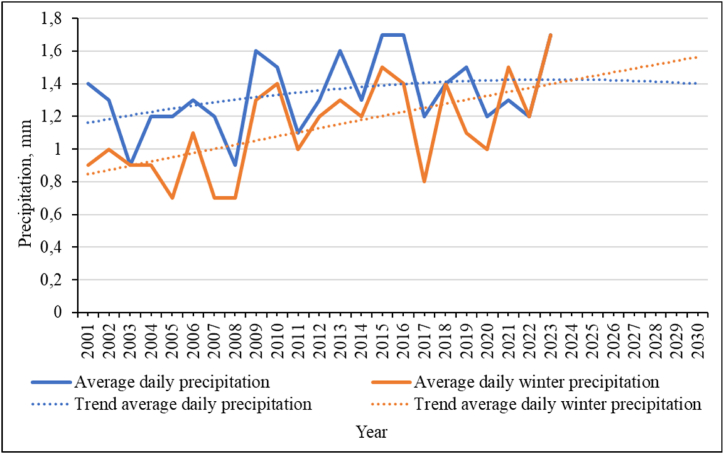
Average daily precipitation for the study area.

According to the data presented in Fig. 13, there is a tendency to increase the amount of precipitation per day, both in general throughout the year and in winter. Although the trend line for the polynomial dependence of the second degree for the average values for the year shows a downward trend. This may be due to the redistribution of precipitation between summer and winter. The amount of precipitation falling per day in winter will increase, this is clearly visible on the second trend line. This shows that the number of days with heavy rainfall at the same time is increasing, which in winter becomes one of the decisive factors leading to an increase in avalanche hazard.

The regression equations and the confidence value of the approximation (R^2^) of the obtained trend values are given in equation (4) for the average values for the year and equation (5) for the winter period:

(4) $$\begin{array}{c} y=-0,0005x^{2}+0,025x+1,1\\ R2=0,14 \end{array}$$

(5) $$\begin{array}{c} y=-0,00005x^{2}+0,026x+0,8\\ R^{2}=0,36 \end{array}$$

**Fig. 14:**
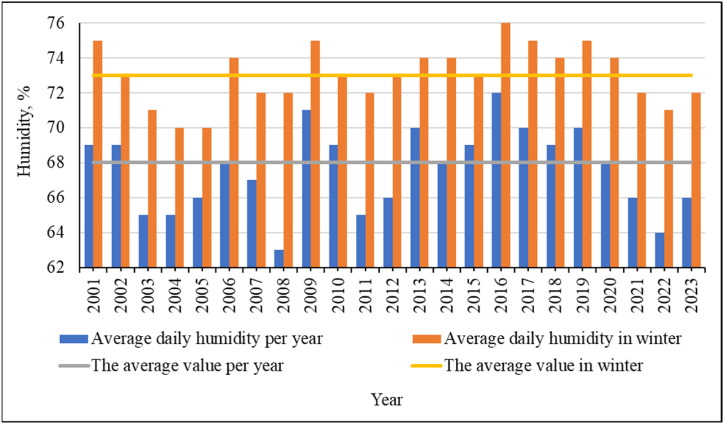
The value of relative air humidity throughout the year and in the winter months, and the forecast of its changes for the study area.

**Fig. 15:**
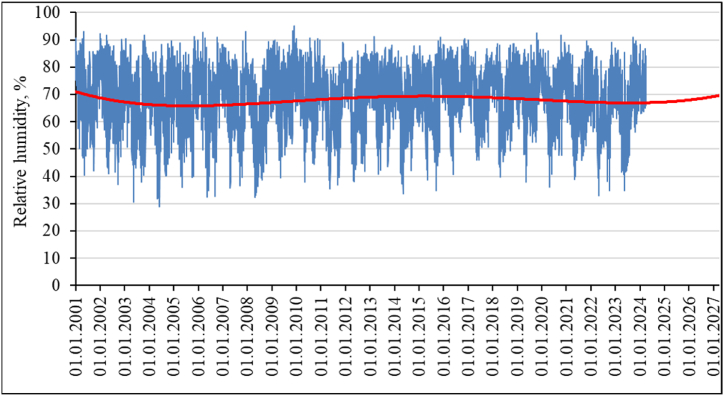
Relative air humidity and the trend of its changes for the study area.

**Fig. 16:**
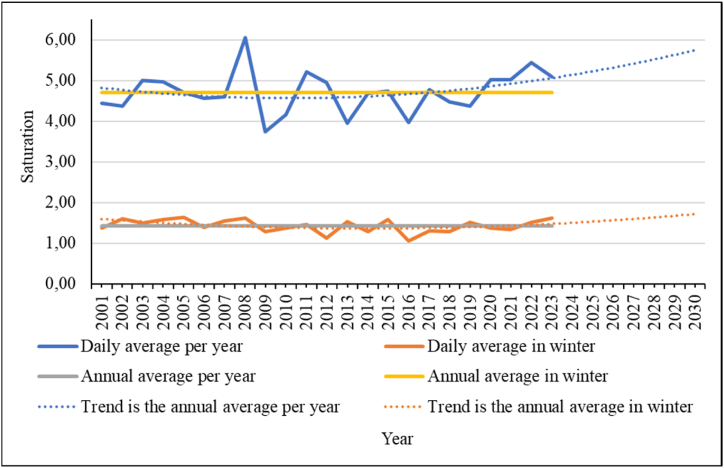
Air saturation deficit and trends in its changes for the study area.

Indicators of relative air humidity (Fig. 14, Fig. 15) and air saturation deficit (Fig. 16) change. These indicators are related to each other, as well as to air temperature and precipitation. On the one hand, the sharply continental climate of East Kazakhstan is not characterized by high air humidity. In recent years, it has been seen that the relative humidity has dropped below the annual average. But the general trend of increasing relative humidity is also visible in Fig. 15, where the trend line is built according to a polynomial dependence of the fourth degree. The confidence value of the approximation for the obtained trend values is R^2^ = 0.013.

The saturation deficit is also an indicator of air humidity. It is defined as the difference between the saturated vapor pressure and the actual vapor pressure in a volume of air. But it is better to say that this indicator characterizes the dryness of the air. The higher the indicator, the drier the air, the more water vapor it can absorb. For the winter period, this indicator is not of great importance, since its value in the cold period does not rise above 2. Cold air cannot contain a large amount of water vapor. But you should pay attention to trends in possible changes in the future. As the air temperature rises, including in winter, the saturation deficit will increase (Fig. 16).

The regression equations and the confidence value of the approximation (R^2^) of the obtained trend values are given in equation (6) for the average values for the year and equation (7) for the winter period:

(6) $$\begin{array}{c} y=0,003x^{2}-0,06x+4,9\\ R^{2}=0,07 \end{array}$$

(7) $$\begin{array}{c} y=0,0014x^{2}-0,04x+1,6\\ R^{2}=0,17 \end{array}$$

As we can see from the graphs presented on changes in air temperature, precipitation, relative humidity and lack of air saturation, the microclimate of the territory is changing, and these trends will continue in the coming years. The climate will become more humid and warmer, which will inevitably affect the avalanche-prone situation in the region.

**Fig. 17:**
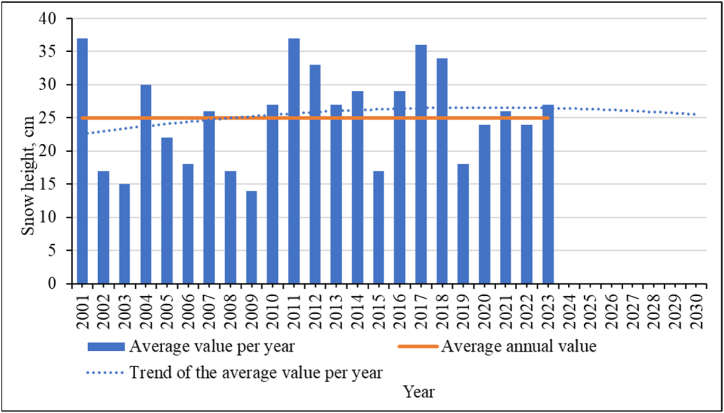
Snow depth and trend for the study area.

All these conditions will affect the formation of snow cover. The average values of the snow cover height for each year are shown in Fig. 17. There is a tendency to exceed the average annual level. At the same time, the trend line shows a tendency to decrease the average height of the snow cover. This can be attributed to an increase in air temperature, including in winter, which will lead to snow melting, its compaction, and crystallization processes. All this is also an additional factor in increasing the level of avalanche hazards in the region.

The regression equation and the confidence value of the approximation (R^2^) of the obtained trend values are given in equation (8):

(8) $$\begin{array}{c} y=-0,011x^{2}+0,44x+22,2\\ R^{2}=0,032 \end{array}$$

**Fig. 18:**
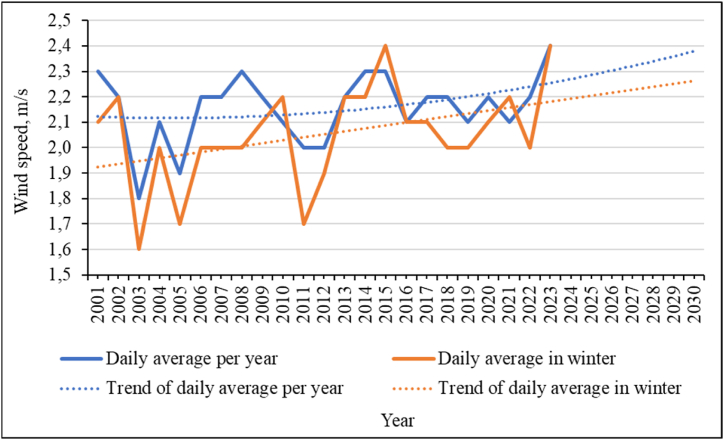
Wind speed and trends in its changes for the study area.

A very important factor influencing avalanches is the wind regime of the area. According to the national meteorological service of Kazakhstan « Kazhydromet», in Kazakhstan as a whole, the average annual wind speed decreases slightly over time. Wind speed extremes also decrease in severity. But, if we consider the wind speeds for the study area, we will see that there is a tendency for them to increase (Fig. 18). This trend is relevant both in general for the average values for the year and for the average values of wind speed in winter. The wind in the mountainous region leads to the formation of visors and marks on the slopes. Subsequently, the collapse of these structures leads to avalanches. An increase in wind speed will lead to an increase in local changes in snow cover on the slopes and, accordingly, to an increase in avalanche hazards in the region.

The regression equations and the confidence value of the approximation (R^2^) of the obtained trend values are given in equation (9) for the average values for the year and equation (10) for the winter period:

(9) $$\begin{array}{c} y=0,0004x^{2}-0,004x+2,1\\ R^{2}=0,1025 \end{array}$$

(10) $$\begin{array}{c} y=0,012x+1,9\\ R^{2}=0,1632 \end{array}$$

**Fig. 19:**
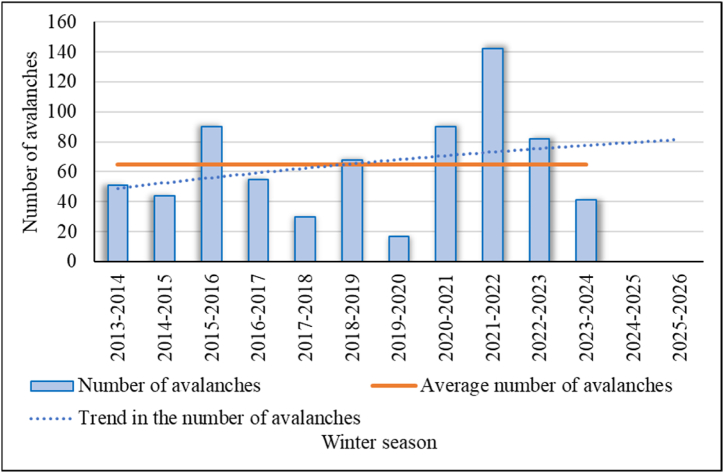
The number of avalanches in East Kazakhstan region for various winter seasons.

**Fig. 20:**
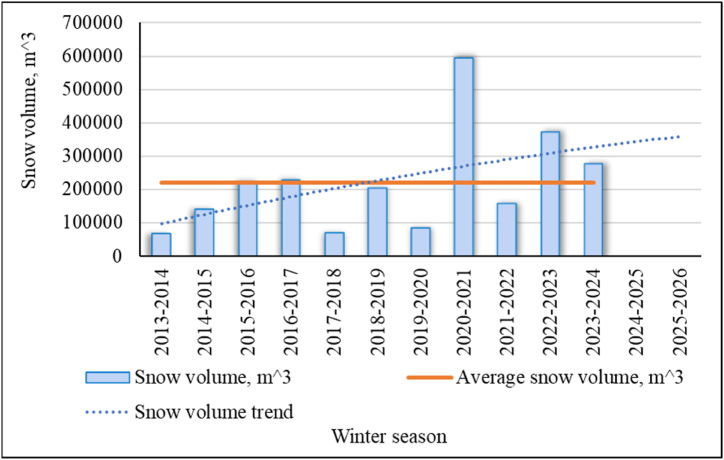
Snow volume of avalanches in East Kazakhstan region for various winter seasons.

Fig. 19, Fig. 20 show generalized statistics on the number and volume of avalanches in East Kazakhstan region for various winter seasons since 2013 (earlier statistics are not available). We can see from these drawings that the winters of 2020–2021 and 2021–2022 were critical for the region. The 2022–2023 and 2023–2024 seasons were also above average. Observations of the beginning of the 2024–2025 season show that in some areas of the region, a monthly snowfall rate fell in early November 2024. The forecast for the coming winter according to the trend lines also shows an increase in the level of avalanche hazard in the region (R^2^ = 0,07; 0,24).

This forecast is quite consistent with the climatic changes that we have identified when reviewing meteorological data. We see a connection between the general trends of increasing air temperature, precipitation and wind speed in winter and an increase in the number of avalanches and their volumes in recent years in East Kazakhstan.

### 4.2 Relationship between weather conditions and spontaneous avalanches

We assume that parameters such as wind speed, precipitation and air temperature affect spontaneous avalanches. With the help of modeling, we analyzed 5 avalanche-prone areas.

To study the possibility of making probabilistic forecasts of avalanches in the study area, data on meteorological conditions and spontaneous snow avalanches in avalanche-prone areas Bogatyrevskaya site, Prokhodnaya, Sogornoye-Barlyk, Tainty and Pikhtovka. For comparative analysis, data on basic meteorological conditions were taken 3 days before, on the day of, and after the date of spontaneous avalanches.

For the Bogatyrevskaya site, the date for spontaneous avalanches was chosen – 07.01.2023.

**Fig. 21:**
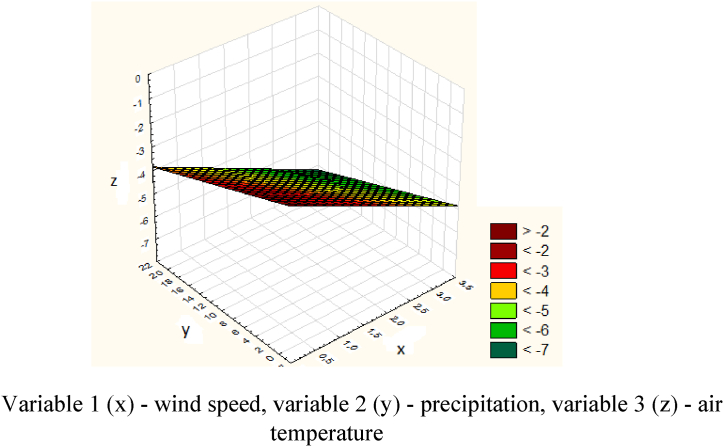
Graph of the dependence of air temperature, wind speed and precipitation for the Bogatyrevskaya site. Variable 1 (x) - wind speed, variable 2 (y) - precipitation, variable 3 (z) - air temperature.

Table 2 shows data for the avalanche-prone area - Bogatyrevskaya. Next, the data obtained for the Bogatyrevskaya site was processed in the Statistica program (Fig. 21).

**Table 2:** Meteorological data for the Bogatyrevskaya site.

Date	Wind speed (x), m/s	Precipitation (y), mm	Air temperature (z), ^о^С
04.01.2023	3	0.4	−4.9
05.01.2023	2	19.7	−3.9
06.01.2023	0.3	16.6	−3.2
07.01.2023	0.5	4.6	−1.6
08.01.2023	1.6	3	−5
09.01.2023	1	2.3	−1.1
10.01.2023	0.8	14.1	−6.9

The model of the dependence of air temperature, wind speed and precipitation for the Bogatyrevskaya site is expressed by the following equation (11):

(11) z=−1.6846−0.9627x−0.098y

For the Prokhodnaya section, the date of the last spontaneous avalanche was chosen – 09.01.2019.

Table 3 shows data for the avalanche-prone area - Prokhodnaya.

**Table 3:** Meteorological Data for the Prohodnaya section.

Date	Wind speed (x), m/s	Precipitation (y), mm	Air temperature (z), ^о^С
06.01.2019	0	–	−25.7
07.01.2019	6.3	–	−8.7
08.01.2019	4.5	–	−8
09.01.2019	5.8	1.1	−0.4
10.01.2019	1.8	8.4	−3.2
11.01.2019	0	1.8	−13.4
12.01.2019	0	1.5	−13.1

**Fig. 22:**
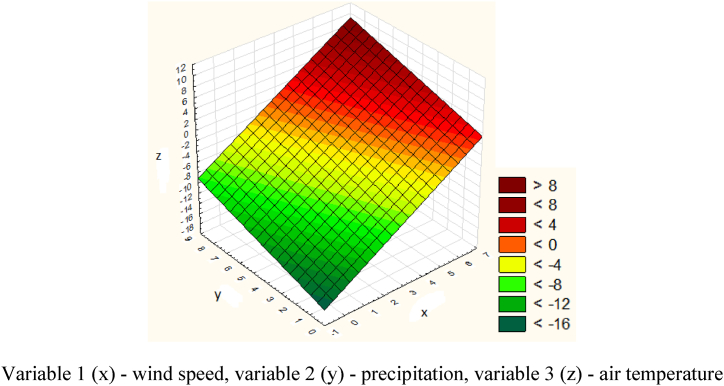
Graph of the dependence of air temperature, wind speed and precipitation in the Prokhodnaya section. Variable 1 (x) - wind speed, variable 2 (y) - precipitation, variable 3 (z) - air temperature.

Further, the obtained data for the Prokhodnaya section were also processed in the Statistica program (Fig. 22).

The causes of spontaneous avalanches were increased air temperature, heavy rainfall and increased wind. The descent point along the road is 34,650 m. The volume of snow is 4300 m^3^. Rolling out of snow mass - complete blocking of the road. There were no casualties or destruction.

The model of dependence of air temperature, wind speed and amount of precipitation on the Prokhodnaya section is expressed by the following equation (12):

(12) z=−14.6872+2.2983x+0.8736y

For the Sogornoye-Barlyk section, the date of the last spontaneous avalanche was chosen – 25.11.2021.

Table 4 shows data for the avalanche-prone area - Sogornoye-Barlyk.

**Table 4:** Meteorological Data for the Sogornoye-Barlyk section.

Date	Wind speed (x), m/s	Precipitation (y), mm	Air temperature (z), ^о^С
22.11.2021	8.4	–	0.5
23.11.2021	2.5	3.4	−4.4
24.11.2021	1.4	10.9	−1.9
25.11.2021	4.5	–	1.9
26.11.2021	3.5	3.8	−4
27.11.2021	3.3	–	−17.8
28.11.2021	6.1	–	−11.4

**Fig. 23:**
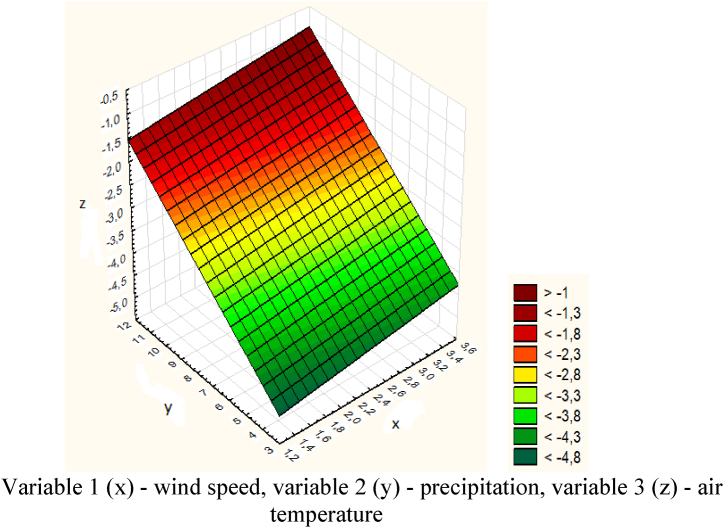
Graph of temperature, wind speed and precipitation for the Sogornoye-Barlyk section. Variable 1 (x) - wind speed, variable 2 (y) - precipitation, variable 3 (z) - air temperature.

Further, the obtained data for the Sogornoye-Barlyk section was also processed in the Statistica program (Fig. 23).

The causes of spontaneous avalanches were increased temperatures and rainfall. The descent point along the road is 14,050 m. The volume of snow is 250 m^3^. Rolling out of snow mass - complete blocking of the road. There were no casualties or destruction.

The model of the dependence of temperature, wind speed and precipitation for the Sogornoye-Barlyk section is expressed by the following equation (13):

(13) z=−6.2887+0.2519x+0.370y

For the Tainty section, the date of the last spontaneous avalanche was chosen – 23.01.2020.

**Fig. 24:**
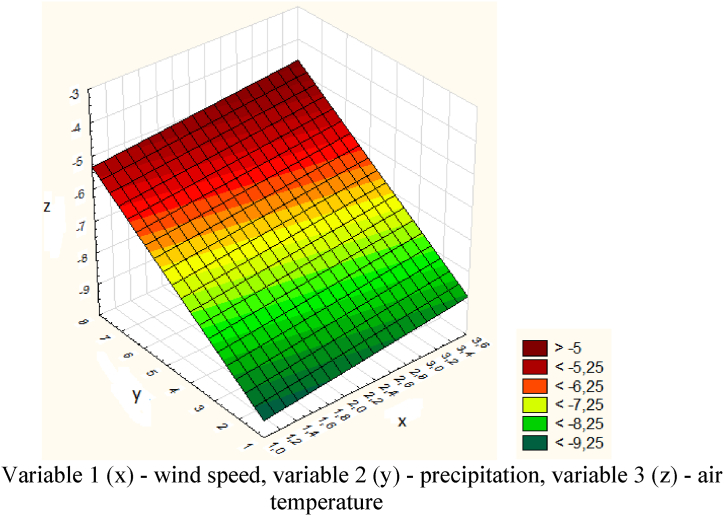
Graph of temperature, wind speed and precipitation for the Tainty section. Variable 1 (x) - wind speed, variable 2 (y) - precipitation, variable 3 (z) - air temperature.

Table 5 shows data for the avalanche-prone area - Tainty. Further, the obtained data for the Tainty site were also processed in the Statistica program (Fig. 24).

**Table 5:** Meteorological Data for the Tainty site.

Date	Wind speed (x), m/s	Precipitation (y), mm	Air temperature (z), ^о^С
20.01.2020	1.1	1.7	−8.7
21.01.2020	2.1	5.9	−8.2
22.01.2020	3.4	6.7	−6.6
23.01.2020	3	4.9	−6.2
24.01.2020	1.6		−7.5
25.01.2020	2.5	7.4	−4
26.01.2020	3	6.7	−4.6

The causes of spontaneous avalanches were heavy snowfall, increased wind and poor visibility. The descent point along the road is 100 m. The volume of snow is 1100 m^3^. There were no casualties or destruction.

The model of the dependence of temperature, wind speed and precipitation over the Tainty site is expressed by the following equation (14):

(14) z=−10.3211+0.2617x+0.5909y

For the Pikhtovka section, the date of the last spontaneous avalanche was chosen – 25.12.2015.

**Fig. 25:**
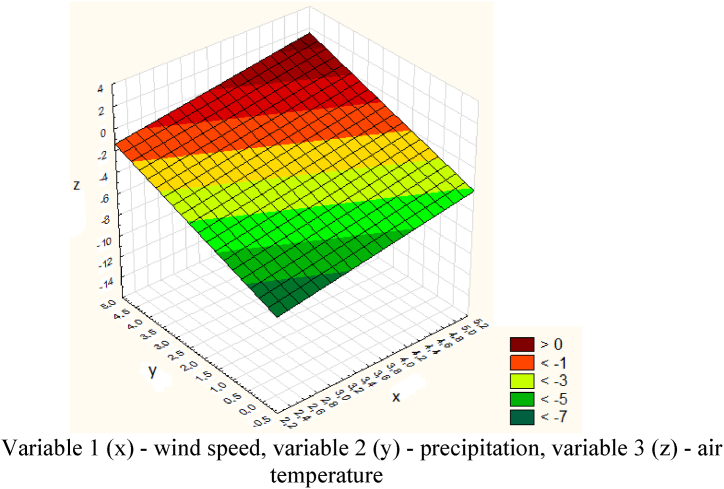
Graph of temperature, wind speed and precipitation in the Pikhtovka area. Variable 1 (x) - wind speed, variable 2 (y) - precipitation, variable 3 (z) - air temperature.

Table 6 shows data for the avalanche-prone area Pikhtovka. Next, the obtained data for the Pikhtovka site was processed in the Statistica program (Fig. 25). The model of the dependence of temperature, wind speed and precipitation in the Pikhtovka area is expressed by the following equation (15):

(15) z=−8.687+0.9804x+1.027y

**Table 6:** Meteorological Data for the Pikhtovka site.

Date	Wind speed (x), m/s	Precipitation (y), mm	Air temperature (z), ^о^С
22.12.2015	2.4	0.2	−14.2
23.12.2015	5	1.4	−8.5
24.12.2015	3.3	2.2	−2.3
25.12.2015	2.6	4.4	−0.2
26.12.2015	3	0.3	1.3
27.12.2015	4.5	0	0.9
28.12.2015	4.3	0	−0.1

The causes of spontaneous avalanches were heavy snowfall, increased wind and poor visibility. Landing point along the road – 62,650 m. Snow volume – 500 m^3^. There were no casualties or destruction.

It should be noted that all spontaneous avalanches occurred in the winter months, except for the November date of the last spontaneous avalanche in the Sogornoye-Barlyk section. November is considered a winter month for the sharply continental climate of the East Kazakhstan region.

Considering changes in air temperature in the days preceding an avalanche, one can see a main trend that manifests itself regardless of the year and month of the spontaneous avalanche. A sharp warming during the three days before the avalanche led to a spontaneous avalanche.

An increase in air temperature is still not the only condition for a spontaneous avalanche. In the days preceding the avalanche, the wind speed gradually increased. The condition that influenced the spontaneous avalanche is also precipitation, in most cases it was heavy snowfall. Rainfall also contributed to the melting of snow from the slope.

## 5 Conclusion

The study examined meteorological and snow-measuring data on 497 avalanche-prone areas of the East Kazakhstan region. 325 avalanche-prone areas pose a threat to infrastructure facilities and people's lives. Based on the analysis, the 10 most dangerous sites were identified. Meteorological data from 7 weather stations are collected here for further detailed avalanche monitoring.

The study carried out a comprehensive analysis of data from meteorological stations and snow measuring routes. These data cover the period from 2001 to the present. The aim was to identify patterns in changing climatic conditions and their impact on the avalanche situation.

The analysis of climate change in the region showed the following results:

- the average annual air temperature in the studied region shows a steady upward trend. This is confirmed by the polynomial dependence model of the second degree. The winter of 2023–2024 was the warmest during the entire observation period;
- the average annual precipitation is also increasing, especially noticeable in winter. This leads to an increase in the number of days with intense precipitation and an increased risk of avalanches;
- the wind speed in the studied area, in contrast to the national trend, is increasing. This can contribute to the formation of snow canopies on the slopes;
- the relationship between the increasing trend in the number of avalanche incidents and the volume of avalanches in the region with ongoing climatic changes has been revealed;
- the general patterns determining the conditions of avalanche formation in avalanche-prone areas of East Kazakhstan region are revealed;
- real cases of spontaneous avalanches with an analysis of meteorological conditions are considered for each site. This made it possible to build probabilistic models of avalanche events. Data analysis has shown that the main factors contributing to avalanches are a sharp increase in air temperature, increased wind speed and heavy precipitation.
- the approaches that were used to collect data necessary to create a system for monitoring and forecasting avalanche activity are considered. Information objects have been identified that describe data sets that allow monitoring and forecasting of avalanche activity in the region.

Further research will be aimed at creating an avalanche monitoring system in the region and integrating data into a single database to improve the effectiveness of avalanche hazard forecasts. At the same time, considering regional climate changes is an important task that requires an integrated approach and the use of modern technologies. This approach will improve the accuracy of forecasts and the effectiveness of measures to protect people and infrastructure from the avalanche threat.

In addition, to increase the effectiveness of the monitoring system, it is important to develop integrated solutions that will include data from various sensors installed directly in avalanche-prone areas. The inclusion in the system of data on snow cover, air temperature at various altitudes, wind speed and other meteorological factors recorded in real time will make monitoring more accurate and operational. This will create conditions for the creation of an early warning system for possible avalanches.

Research in the field of avalanche hazard in East Kazakhstan is of great importance for improving the safety of mountain areas and suggests wide opportunities for further scientific research and practical application at the international level.

## CRediT authorship contribution statement

**Olga Petrova:** Writing – review & editing, Writing – original draft, Methodology, Investigation, Formal analysis, Conceptualization. **Natalya Denissova:** Writing – review & editing, Writing – original draft, Methodology, Investigation, Formal analysis, Conceptualization. **Gulzhan Daumova:** Writing – review & editing, Writing – original draft, Methodology, Investigation, Formal analysis, Conceptualization. **Yelena Ivashchenko:** Resources, Investigation, Data curation. **Evgeny Sergazinov:** Resources, Investigation, Data curation.

## Ethics declaration

Review and/or approval by an ethics committee as well as informed consent was not required for this study because this article did not involve any direct experimentation/studies on living beings.

## Data availability statement

The data presented in this research are available on request.

## Funding

The article presents the results of scientific research obtained during the implementation of a scientific and technical program BR21882022 on the topic: “Research of avalanche activity in the East Kazakhstan region for development of monitoring systems and scientific substantiation of their placement” within the framework of program-targeted financing.

## Declaration of Competing Interest

The authors declare that they have no known competing financial interests or personal relationships that could have appeared to influence the work reported in this paper.
